# Decreased Circulating Levels of Dickkopf-1 in Patients with Exudative Age-related Macular Degeneration

**DOI:** 10.1038/s41598-017-01119-2

**Published:** 2017-04-28

**Authors:** Fangfang Qiu, Zhen Liu, Yueping Zhou, Jia He, Songjian Gong, Xue Bai, Yingxia Zeng, Zuguo Liu, Jian-xing Ma

**Affiliations:** 1 Eye Institute of Xiamen University, Fujian Province Key Laboratory of Ophthalmology and Visual Science, 4th Floor, Chengyi Building, Xiang’an Campus of Xiamen University, Xiang’an South Road, Xiamen Fujian, 361102 China; 2 Department of Physiology, The University of Oklahoma Health Sciences Center, Oklahoma City, Oklahoma 73104 United States of America; 3 Affiliated Xiamen Eye Center of Xiamen University, Xiamen Fujian, China; 4 Department of Ophthalmology, Affiliated Zhongshan Hospital of Xiamen University, Xiamen Fujian, China

## Abstract

Aberrant activation of the Wnt/β-catenin signaling pathway plays a pathogenic role in retinal inflammation and neovascularization. Here, we investigated whether circulating levels of Dickkopf-1 (DKK-1), a specific inhibitor of this pathway, are altered in patients with exudative age-related macular degeneration (AMD). Plasma was obtained from 128 patients with exudative AMD, 46 patients with atrophic AMD and 111 healthy controls. DKK-1 levels in plasma were measured using ELISA, and data analyzed with one-way ANOVA, logistic regression analysis and receiver-operating characteristic analysis (ROC). We found that DKK-1 levels were decreased in exudative AMD patients, compared with healthy controls (*P* < 0.001) and atrophic AMD patients (*P* < 0.001). The decrease was more prominent in patients with classic choroidal neovascularization (CNV) than those with occult CNV (*P* < 0.001). The odds ratio (OR) of exudative AMD was 11.71 (95% CI; 5.24–6.13) for lowest versus upper quartile of DKK-1 levels. For discriminating exudative AMD patients, the optimum diagnostic cutoff of DKK-1 was 583.1 pg/mL with the area under curve (AUC) 0.76 (95% CI, 0.70–0.82; *P* < 0.001), sensitivity 78.1% and specificity 63.1%. These findings suggested that decreased circulating DKK-1 levels are associated with the development and severity of exudative AMD, and have potential to become a biomarker for exudative AMD.

## Introduction

Age–related macular degeneration (AMD) is the leading cause of severe and irreversible vision loss in elderly population in developed countries^1, 2^. There are two forms of this progressive disease: atrophic AMD and exudative AMD. Exudative AMD, an advanced stage of AMD, is responsible for 90% severe vision loss in AMD patients^2^. Choroidal neovascularization (CNV), defined as newly formed blood vessels arising from choriocapillaries, is the hallmark of exudative AMD and the main cause of vision loss^3^. Atrophic AMD can progress to exudative AMD^4^. The pathogenesis of exudative AMD is not fully understood, and biomarkers for its clinical detection are limited. It is, therefore, essential and important to identify etiology and the risk factor(s) associated with exudative AMD and to develop novel biomarkers for detection and prognosis of this disease.

The Wnt/β-catenin signaling pathway is a multifunctional pathway that is involved in embryonic development and also controls homeostatic self-renewal in various adult tissues^5^. Wnt proteins are a family of secreted glycoproteins which bind to a receptor complex consisting of frizzled and low-density lipoprotein receptor-related protein 5/6 (LRP5/6), leading to the stabilization and accumulation of β-catenin. Subsequently, β-catenin translocates into the nucleus and activates transcription of multiple target genes including some inflammatory and angiogenic factors^6, 7^. Dickkopf-1 (DKK-1), a well-established natural inhibitor of Wnt signaling, binds to LRP5/6 and sequesters LRP5/6 from dimerization with frizzled, subsequently inhibiting Wnt signaling^7^.

DKK-1 is a secreted protein and readily detectable in the circulation, and measurement of its circulating levels is useful for the clinical investigation of the diseases associated with Wnt signaling^8^. The DKK-1 level has been proposed as a diagnostic and prognostic biomarker for many diseases such as hepatocellular carcinoma^9^, pancreatic cancer^10^, lung and esophageal cancer^11^. Our recent study also found that changes of DKK-1 levels in the circulation are associated with the development of diabetic retinopathy^12^, in which over-activation of the Wnt pathway plays a pathogenic role. Our previous studies, based on cell culture and the laser-induced CNV animal model, demonstrated that the Wnt/β-catenin signaling pathway plays an important role in the development of CNV. This pathway is activated in the retina and retinal pigment epithelial cells (RPE) in the CNV mouse model^13^, and activation of the Wnt/β-catenin pathway plays a pathogenic role in the up-regulation of angiogenic factors^7^. However, there is no clinical evidence implicating the dysregulation of Wnt signaling in exudative AMD in human patients. In order to better understand the role of the Wnt pathway in the pathogenesis of AMD and identify a biomarker for this disease, we measured plasma DKK-1 levels in exudative AMD patients and investigated if circulating DKK-1 levels are associated with this disease in the present study. We also determined the correlation of DKK-1 levels with clinical profiles, such as subtype of CNV, severity of the disease, and analyzed the potential value of circulating DKK-1 levels as a biomarker for exudative AMD.

## Results

### Demographic characteristics of subjects

Characteristics of subjects were shown in Table 1. A total of 285 subjects including 111 healthy controls, 46 patients with atrophic AMD and 128 patients with exudative patients were recruited. Subjects were gender and age-matched among these groups. There were also no differences in smoking status, hypertension, diabetes or cardiovascular diseases among these subjects. Of patients with exudative AMD, there were 47 patients with classic CNV and 81 with occult CNV; 35 patients had bilateral exudative AMD, and 93 had unilateral exudative AMD.

**Table 1:** Demographic and Clinical Characteristics of Subjects.

Characteristic	Healthy controls (n = 111)	Atrophic AMD (n = 46)	Exudative AMD (n = 128)
Sex, n (%)			
Female	56 (50.5)	26 (56.5)	68 (53.1)
Male	55 (49.5)	20 (43.5)	60 (46.9)
Age (years)			
Mean ± SD	64.99 ± 8.47	65.67 ± 8.55	66.18 ± 8·30
Median (range)	68 (50–84)	68 (50–80)	68 (50–88)
Smoking, n (%)			
Never	76 (68.5)	30 (65.2)	77 (60.2)
Former	17 (15.3)	8 (17.4)	23 (17.9)
Current	18 (16.2)	8 (17.4)	28 (21.9)
Systemic diseases, n (%)			
Hypertension	31 (27.9)	12 (26.1)	38 (29.7)
Cardiovascular disease	6 (5.4)	3 (6.4)	8 (6.2)
Subtypes of CNV, n			
Classic CNV			47
Occult CNV			81
Laterality of exudative AMD, n			
Unilateral			93
Bilateral			35

AMD = age-related macular degeneration; CNV =choroidal neovascularization.

Data were expressed as number (%), mean ± SD, or median (range).

Chi-square tests were used for comparisons of the categorical variables; One-way analysis of variance (ANOVA) was used for comparisons of the continuous variable (age).

All *P* > 0.05 for differences of sex, age, smoking status, hypertension and cardiovascular disease among healthy controls, atrophic AMD and exudative AMD.

### Plasma levels of DKK-1 were decreased in patients with exudative AMD

**Figure 1:**
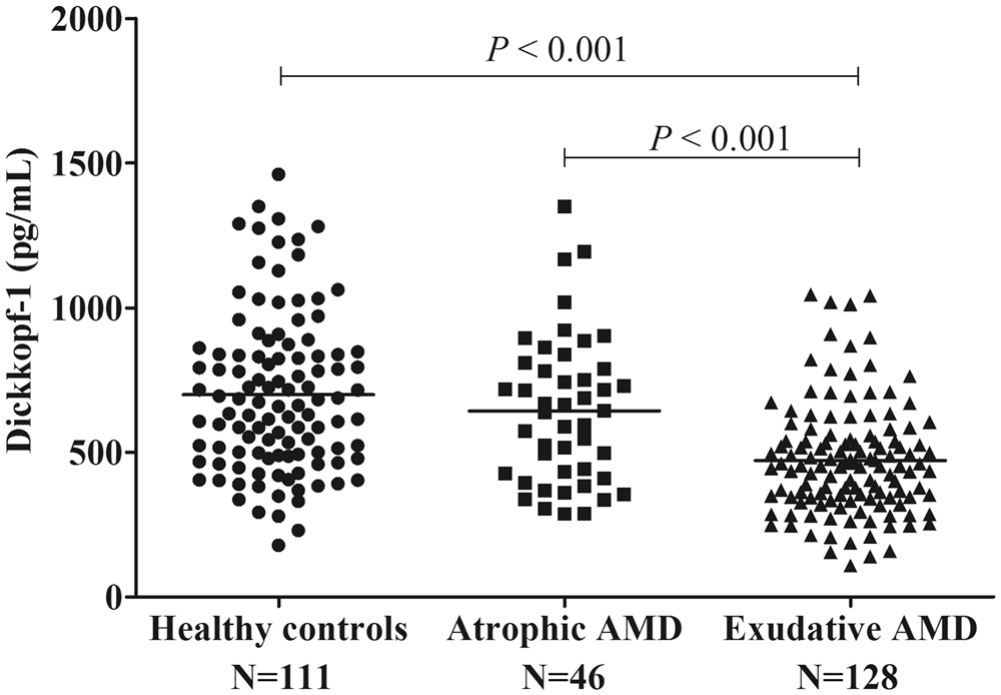
Plasma Dickkopf-1 (DKK-1) Levels were Decreased in Exudative Age-Related Macular (AMD), Compared with Healthy Controls and Patients with Atrophic AMD. Analysis was performed by one-way analysis of variance with post hoc Tamhane tests. Each spot represents one patient. Middle lines represented the mean.

The distributions of plasma DKK-1 levels in healthy controls, patients with atrophic AMD and patients with exudative AMD were all approximately normal distribution (One-sample Kolmogorov–Smirnov test, all *P* > 0.05; Table 2). Depending on the normal distribution of DKK-1 levels, one-way ANOVA analysis was used to analyze the difference of DKK-1 among these study groups. It was found that there were a significant difference of DKK-1 levels among these three groups (one-way ANOVA analysis, F value = 28.98; *P* < 0.001; Table 2, Fig. 1). Mean DKK-1 levels in patients with exudative AMD were decreased by 32.5% and 26.7%, respectively, compared with those in healthy controls (Tamhane test, *P* < 0.001) and patients with atrophic AMD (Tamhane test, *P* < 0.001). However, no significant difference was observed in DKK-1 levels between patients with atrophic AMD and healthy controls (Tamhane test; *P* = 0.533). These results suggested that decreased levels of DKK-1 in the circulation are associated with exudative AMD, but not atrophic AMD.

**Table 2:** Normal Distribution of Dickkopf-1 Levels in Study Subjects.

Group	N	Dickkopf-1 Levels (pg/mL)			Normal distribution tested^†^	
		Mean ± SD	Minimum	Maximum	Z values	P values
Healthy controls	111	699.21 ± 272.99	179.45	1460.77	0.856	0.456
Atrophic AMD	46	643.61 ± 253.22	287.86	1351.23	0.601	0.862
Exudative AMD						
Total	128	471.78 ± 192.39	108.37	1045.14	1.178	0.124
Unilateral CNV	93	479.95 ± 200.38	108.37	1045.14	1.111	0.169
Bilateral CNV	35	450.09 ± 170.13	158.74	896.78	0.751	0.626
Occult CNV	81	504.52 ± 202.73	139.85	1045.14	1.131	0.155
Classic CNV	47	415.37 ± 159.93	108.37	785.80	0.615	0.843

AMD = age-related macular degeneration; CNV = choroidal neovascularization.

^†^Data were tested by one-sample Kolmogorov–Smirnov test and were considered normal distribution when *P* > 0.05.

### Decreased plasma DKK-1 levels were associated with the subtype of CNV in exudative AMD

**Figure 2:**
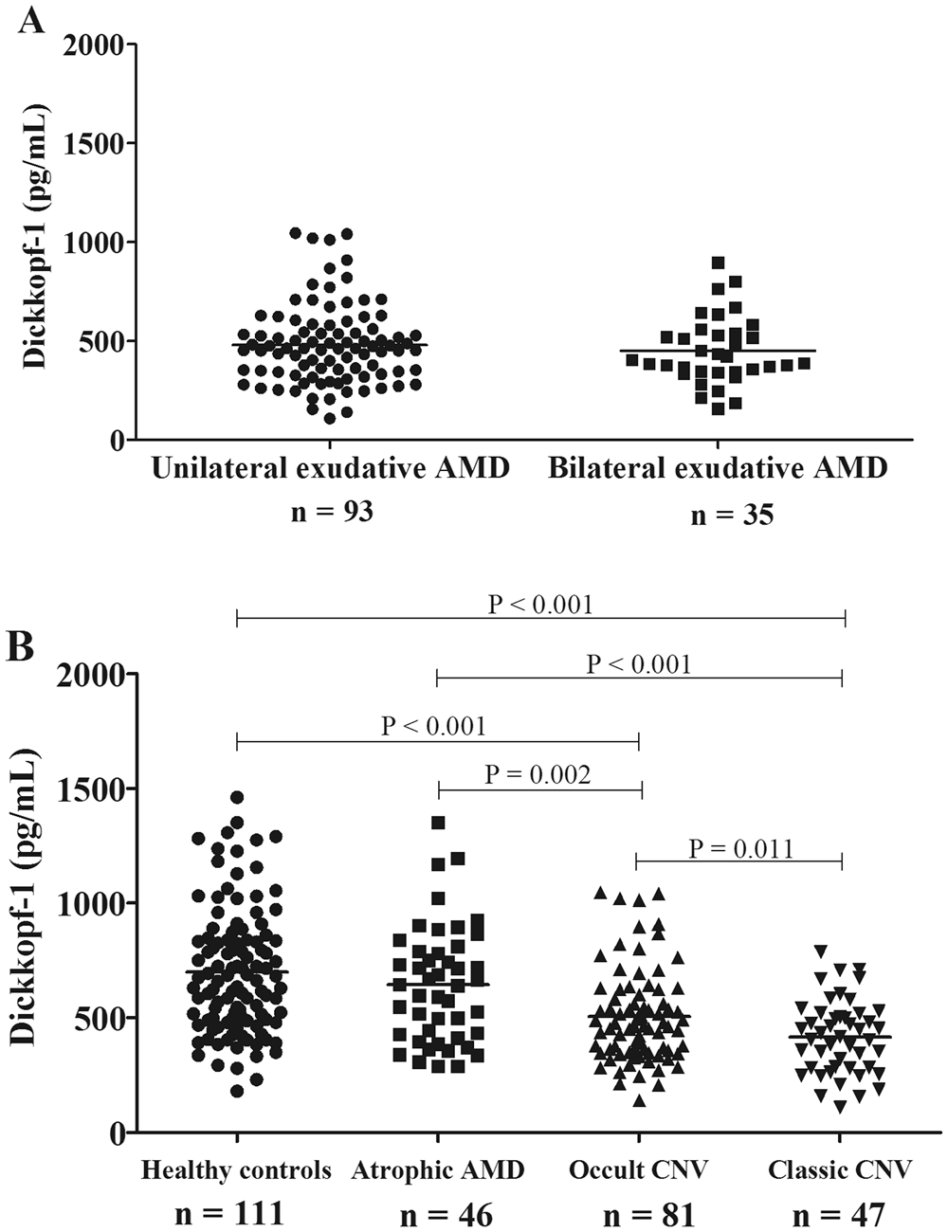
Lower Plasma Dickkopf-1 (DKK-1) Levels were Associated with Severity of Exudative Age-Related Macular Degeneration (AMD). (**A**) Comparison of DKK-1 levels between patients with unilateral exudative AMD and bilateral exudative AMD; (**B**) Comparison of DKK-1 levels between patients with occult CNV and classic CNV. Data were analyzed by Student’s t-test. Each spot represents one patient. Middle lines represented the mean.

We further investigated the relations between plasma DKK-1 levels and the progression or severity of exudative AMD. Patients with exudative AMD were divided into unilateral exudative AMD and bilateral exudative AMD according to laterality of having exudative AMD. They were also divided into occult CNV and classic CNV according the subtypes of CNV. The distributions of plasma DKK-1 levels in these groups were all approximately normal distribution (One-sample Kolmogorov–Smirnov test, all *P* > 0.05; Table 2). Depending on the normal distribution of DKK-1 levels, Student t-test was used to analyze the difference of DKK-1 levels between two groups. No significant difference was found between the patients with unilateral exudative AMD and patients with bilateral exudative AMD (Student t-test, *P* = 0.55; Table 2, Fig. 2A). However, it was found that DKK-1 levels were significantly lower in patients with classic CNV, than in those with occult CNV (Student t-test, *P* = 0.011; Table 2; Fig. 2B), and DKK-1 levels in both occult CNV and classic CNV patients were decreased compared with that in healthy control and atrophic AMD (Student t-test, all *P* < 0.01; Table 2; Fig. 2B). Classic CNV and occult CNV are two subtypes of CNV in exudative AMD. Occult CNV is believed to be located in the sub-RPE space, while classic CNV is believed to be located in the subretinal space, and progressive leakage can be seen throughout the angiogram, and is associated with more aggression of the disease and more severe vision loss^14^. These results indicated that the reduction of DKK-1 is also associated with the progression or severity of exudative AMD or CNV.

### Decreased plasma DKK-1 levels were associated with the higher risk of exudative AMD

**Figure 3:**
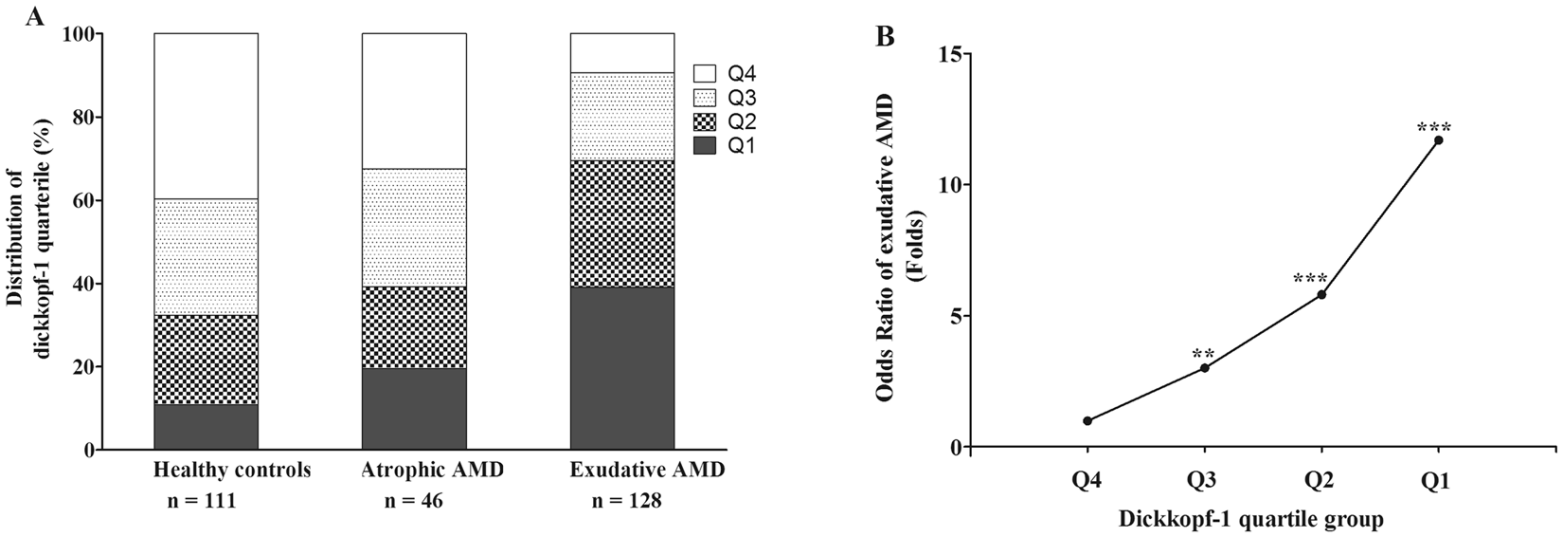
Odds ratios (OR) of exudative age-related macular degeneration (AMD) by dickkopf-1 (DKK-1) quartiles. (**A**) Distribution of DKK-1 quartiles in healthy controls, patients with atrophic AMD and exudative AMD. (**B**) OR trend of having exudative AMD by DKK-1 quartiles. Quartiles of DKK-1: Q1, < 393.79; Q2, 393.80~527.73; Q3, 527.74~737.18, and Q4 > 737.19 pg/mL. All comparisons were made to the highest quartile (Q4) of DKK-1 (OR = 1.00) by logistic regression analysis. **P < 0.01; ***P < 0.001.

To further clarify the potential clinical relevance, DKK-1 levels in the study subjects were separated into quartiles. As shown in Table 3 and Fig. 3A, the distribution of quartile of DKK-1 was also found to have significant difference among healthy controls, patients with atrophic AMD, and patients with exudative AMD (*χ* ^2^ = 71.25, *P* < 0.001). Its distribution in exudative AMD was significantly different from that in healthy controls (*χ* ^2^ = 44.44, *P* < 0.001) and patients with atrophic AMD (*χ* ^2^ = 17.78, *P* < 0.001), with the highest proportion of exudative AMD patients (50/128[39.1%]) in the lowest DKK-1 quartile (Q1, <393.79 pg/mL); while the distribution of DKK-1 quartile having no significant difference between atrophic AMD and healthy controls (*χ* ^2^ = 2.36, *P* = 0.50). These results further confirmed that decreased circulating levels of DKK-1 are associated with exudative AMD, suggesting that lower DKK-1 levels may be associated with a higher risk of having exudative AMD.

**Table 3:** Frequencies of Study Subjects According to the DKK-1 Quartile Group.

DKK-1 quartile	Healthy controls n, (%)^‡^	Atrophic AMD n, (%)^‡^	Exudative AMD n, (%)^‡^
Q1	12 (10.81)	9 (19.57)	50 (39.06)
Q2	24 (21.62)	9 (19.57)	39 (30.47)
Q3	31 (27.93)	13 (28.26)	27 (21.09)
Q4	44 (39.64)	15 (32.60)	12 (9.38)
Total	111	46	128

DKK-1 = dickkopf-1; AMD = age-related macular degeneration.

Q1, < 393.79; Q2, 393.80~527.73; Q3, 527.74~737.18, and Q4 > 737.19 pg/mL.

^‡^% of total in quartile.

Values were expressed as the number (%); Chi-square test for trend; *χ* ^2^ = 71.25, *P* < 0.001.

Next, a logistic regression analysis was used to estimate odds ratios (OR) and 95% confidence intervals (95% CI) for the risk of exudative AMD according to the DKK-1 quartile. As shown in Table 4 and Fig. 3B, there was a trend for an increased OR of having exudative AMD with decrease of DKK-1 quartile (*χ* ^2^ = 39.769, P = 0.000): The third versus upper quartile of DKK-1 levels (OR, 3.02, 95% CI [1.38–6.6]; *P* = 0.006); The second versus the upper quartile of DKK-1 levels (OR, 5.81, 95% CI [2.68–12.6]; *P* = 0.000); The lowest versus upper quartile of DKK-1 levels (OR, 11.71, 95% CI [5.24–26.13]; *P* = 0.000). These results suggested that decreased circulating DKK-1 levels were associated with high likelihood or risk of having exudative AMD.

**Table 4:** OR and 95% CI for the Association between Plasma DKK-1 Quartiles with the Prevalence of Exudative AMD.

DKK-1 quartile	β	SE	Wald (*χ* ^2^)	*P* Values	OR (95% CI)
Q4					1.00 (reference)
Q3 versus Q4	1.104	0.400	7.619	0.006	3.02 (1.38–6.61)
Q2 versus Q4	1.760	0.395	19.821	0.000	5.81 (2.68–12.61)
Q1 versus Q4	2.460	0.410	36.046	0.000	11.71 (5.24–26.13)

OR = Odds Ratios; CI = 95% Confidence Intervals; DKK-1 = dickkopf-1; AMD = age-related macular degeneration.

Q1, <393.79; Q2, 393.80~527.73; Q3, 527.74~737.18, and Q4 > 737.19 pg/mL.

Unconditional logistic regression analysis.

### Potential of the circulating DKK-1 levels to become a biomarker for detection of exudative AMD

**Figure 4:**
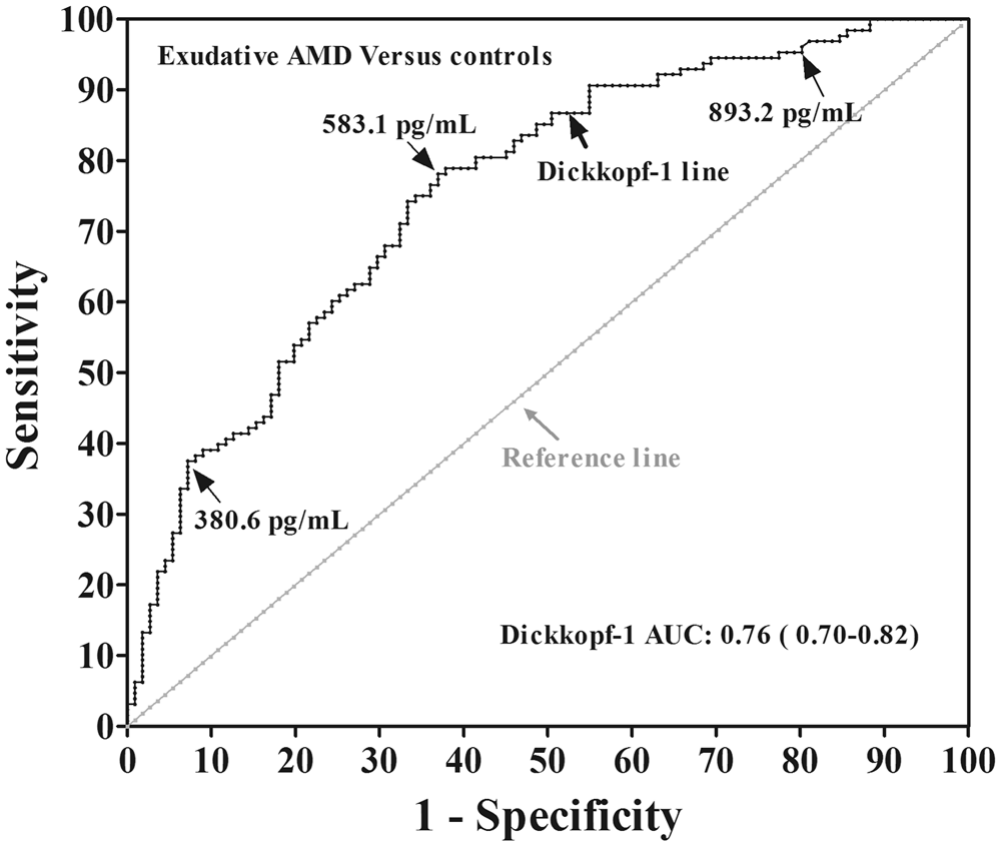
ROC analysis of plasma DKK-1 levels for the diagnosis of exudative AMD. The ROC curve was drawn with the data of 128 exudative AMD patients and 111 healthy controls. Each point on the ROC curve represents the sensitivity vs. False positive rate (1-specifity), corresponding to the cut-off value. ROC = receiver-operating-characteristic curve; AMD = age-related macular degeneration; AUC = area under the ROC curve.

To assess whether circulating DKK-1 levels can be used as a biomarker for detection of exudative AMD, the ROC analysis was performed with DKK-1 levels. Total accuracy was measured by area under the ROC curve (AUC), and optimal cut-off value of DKK-1 was determined by Youden index J. ROC curve showed that the optimum diagnostic cutoff for DKK-1 was 583.1 pg/mL, with an AUC 0.76 (95% CI, 0.70–0.82; *P* < 0.001). This corresponds to a sensitivity of 78.1%, and specificity of 63.1% (Fig. 4). These results suggested that the circulating DKK-1 level has potential to become a biomarker for the detection of exudative AMD.

## Discussion

To our knowledge, this was the first study to investigate circulating DKK-1 levels in patients with AMD. Our results showed that decreased circulating levels of DKK-1 are associated with exudative AMD, but not with atrophic AMD, and the degrees of the DKK-1 decrease are associated with the progression or severity of CNV in exudative AMD; the lower DKK-1 levels in the circulation are associated with the higher likelihood of having exudative AMD. In addition, our study showed that levels of DKK-1 in the circulation have a potential to become a novel biomarker for prediction of exudative AMD.

The Wnt/β-catenin signaling pathway has been shown to be involved in multiple physiological and pathophysiological processes^15^. Dysregulation of the Wnt signaling pathway is known to be associated with a number of human diseases, such as neurodegenerative diseases, neovascular disorders and bone diseases^16–18^. DKK-1 plays important roles in Wnt signaling regulation. DKK-1 expression has been found to be down-regulated in human colon cancer, contributing to activation of the Wnt/β-catenin pathway^19^. Altered DKK-1 levels in the circulation have also been shown to be associated with multiple diseases, in which activity of Wnt signaling is abnormal. Its plasma levels were increased in patients with breast cancer with bone metastases, lung cancer and esophageal cancer^8, 11^. Elevated circulating DKK-1 levels have been associated with reduced progression of radiographic hip osteoarthritis in women^20^. Its levels were decreased in patients with gastric cancer, colorectal cancer, ovarian cancer and cervical adenocarcinoma^21^. Our recent study also found that decreased DKK-1 levels in the circulation are associated with the development of diabetic retinopathy^12^. However, DKK-1 levels in AMD patients have not been previously reported.

The causes of systemic reduction of DKK-1 in exudative AMD are not yet known. It was reported that DKK-1 is expressed at low levels in most normal human tissues^22^, and the circulating DKK-1 is mainly originated from the platelets^23^. Our previous study showed that DKK-1 levels in the vitreous are substantially lower than in the plasma^12^. Therefore, it does not seem likely that the decrease of DKK-1 in the circulation is due to reduced DKK-1 production in the eye. It may be due to the decreased secretion from platelets, in which the mechanism is unknown, requiring further investigation.

Our previous studies revealed that canonical Wnt signaling is over-activated in the eyecup of experimental CNV^13, 24^. We also have confirmed that activation of the Wnt/β-catenin pathway is sufficient to induce retinal inflammation, oxidative stress in cultured ARPE-19 cells and in the retina^7, 24^, and plays a pathogenic role in laser-induced CNV^7, 24^. Kim *et. al* have shown that activation of the Wnt/β-catenin pathway leads to enhanced endothelial cell (EC) proliferation and tube formation through enhancing cyclin E2 expression^25^. Therefore, it is likely that RPE cells and EC represent the primary DKK1 targets. Wnt signaling regulates multiple target genes including angiogenic factors such as VEGF and inflammatory factors such as TNF-α and ICAM-1. VEGF has been shown to play a critical role in the pathogenesis of CNV in AMD^26, 27^. TNF-α also plays a pathogenic role in neovascular AMD^28^. ICAM-1 expression in the RPE plays an important role in leukocyte adherence^29, 30^. As DKK-1 is a natural inhibitor of the Wnt signaling pathway, it is likely that decreased systemic levels of DKK-1 lead to a relatively low DKK-1 concentration in the eye, facilitating the over-activation of the Wnt signaling pathway in the EC in choroidal vessels and RPE, which subsequently leads to secretion of angiogenic factors such as VEGF and PDGF and inflammatory factors such as TNF-α and ICAM-1, and consequently endothelial proliferation, leukocyte adhesion and vascular leakage, promoting the development or progression of CNV. It is also plausible to speculate that individuals with low DKK-1 levels have high risks of developing exudative AMD or accelerated progression of CNV. However, further longitudinal studies are needed to clarify the potential role of decreased DKK-1 levels in the pathogenesis of exudative AMD.

So far, there are only two studies implicating the dysregulation of Wnt signaling pathway in tissues of human with AMD. We recently studied the Wnt signaling in human tissues in patients with AMD, showing aberrant activation of Wnt signaling in the retina and decreased circulating levels of kallistatin, an anti-angiogenic factor with inhibitory an effect on Wnt signaling, in patients with AMD^31^. However, the patients with dry AMD (i.e. atrophic AMD) and exudative AMD were combined, and the data were not analyzed separately. Thus, the study did not address whether the abnormal Wnt pathway is associated with either atrophic AMD or exudative AMD. Another study by Park *et al*.^32^, investigated the association of Wnt signaling modulators, Wnt inhibitory factor 1 (WIF-1) and dickkopf 3 (DKK-3) in aqueous humor of 62 patients with the neovascular AMD (i.e. exudative AMD). However, this study had small sample sizes, and lack disease controls (atrophic AMD). In contrast, our study compared plasma levels of the Wnt signaling inhibitor DKK-1 in exudative AMD to that in healthy controls and disease control (atrophic AMD). In addition, our study analyzed association of decreased DKK-1 levels with odds ratios for risk of exudative AMD, further suggesting a correlation between the DKK-1 levels and exudative AMD, although further longitudinal studies are needed to correlate DKK-1 levels with the course of AMD at different stages in order to determine whether a lower DKK-1 level is a predictive factor for the development of exudative AMD. In brief, our results provide important evidence supporting an association of the Wnt signaling pathway with human exudative AMD.

In clinic, a reliable and low-cost blood marker measurement that is micro-invasive and convenient is preferred for the detection of the development of exudative AMD that needs long-term monitoring and treatment. Some researchers also have attempted to associate some serum cytokines with the development of exudative AMD and evaluated their potential biomarkers for AMD. These cytokines include soluble Flt-1^33^, eotaxin-2^34^, Vitamin D^35^, leptin^36^, and so on. However, so far, no ideal biomarkers are available to detect or characterize exudative AMD. DKK-1 is an excellent candidate for the prediction of exudative AMD because decreases of its levels in the circulation are associated with only the exudative but not atrophic AMD. We attempted to estimate the potential of circulating DKK-1 levels for serving as a biomarker for exudative AMD based on the optimal cut-off values of DKK-1 determined by ROC analysis. The results showed that DKK-1 had a 76% (more than 70%) probability of correctly distinguishing exudative AMD samples from control samples, with a theoretical sensitivity of 78.1% and a specificity of 63.1%, using a cutoff of 583.1 pg/mL. These results suggest that the measurement of plasma DKK-1 levels have potential to be a biomarker for exudative AMD. It is hopefully to improve early diagnostic accuracy for exudative AMD in combination of DKK-1 with circulating cytokines mentioned above, although the specificity 63% is not sufficient for a biomarker test.

Our study has a few limitations. Firstly, because this is a cross-sectional study, it is difficult to determine a cause-effect relationship of decreased DKK-1 levels and the development of exudative AMD, and longitudinal clinical studies are necessary in the future to ascertain whether DKK-1 levels at baseline are predictive of risk of developing exudative AMD and progression of CNV. Secondly, we did not measure circulating levels of other inhibitors of the Wnt pathway. Thirdly, we did not measure DKK-1 levels in local tissues such as CNV membrane and vitreous to assess whether they are correlated with circulating levels. Finally, we did not make a simultaneous measurement of other factors to increase the sensitivity and specificity for prediction of exudative AMD, which remains to be investigated further.

In conclusion, we measured circulating DKK-1 levels in a large group of patients with exudative AMD, atrophic AMD and controls. The results showed an association of decreased DKK-1 levels with the presence or progression of exudative AMD. Further, lower levels of DKK-1 were associated with a higher likelihood of having exudative AMD, and the circulating DKK-1 level has potential to serve as a biomarker for detection and evaluation of exudative AMD. Future prospective investigations should explore whether decreased plasma DKK-1 levels herald the onset of neovascular AMD, why DKK-1 levels decline in these patients, and if interventions that restore normal plasma DKK-1 levels ameliorate the disease and/or improve therapeutic outcomes.

## Methods

### Study design and patients

This case-controlled study was conducted in Affiliated Xiamen Eye Center of Xiamen University, from December, 2009 to September, 2011. Patients with exudative AMD (n = 128), patients with atrophic AMD (n = 46), and healthy controls (n = 111) were enrolled. The study was approved by the Ethics Committee of the center. All methods and analyses were performed in accordance with the approved protocol and guidelines. The written informed consents were obtained from all subjects prior to the study.

The following criteria were used to recruit exudative AMD patients: newly diagnosed; aged 50 years or older. The age-matched controls included two groups, i.e. healthy controls who had no evidence of drusen, pigmentary abnormalities and age-related maculopathy, and disease controls (atrophic AMD). All subjects were Chinese origin and local residents. Subjects were excluded from the study if they had vascularized cornea, poor visualization of fundus and retinal diseases other than AMD, such as high myopia, retinal dystrophies, diabetes or diabetic retinopathy, connective tissue disorder, neoplastic disease, arthritis, bone disease, cognitive decline, Alzheimers disease, neurological disorder and anti-platelet therapy.

Clinical data were collected regarding age, gender, smoking history, history of systemic diseases such as diabetes, heart diseases and previous ocular treatments. Smoking status involved 3 categories: never smoker, current smoker and former smoker. Hypertension was defined as systolic blood pressure ≥140 mmHg, diastolic blood pressure ≥90 mmHg at examination, or diagnosed by a physician previously.

All subjects completed detailed ocular examination including best-corrected visual acuity, slit-lamp biomicroscopy and dilated binocular ophthalmoscopy. In addition, AMD patients underwent color fundus photography, fluorescein angiography (FA), and indocyanine green angiography (ICGA). All of the AMD patients were diagnosed and divided into atrophic AMD and exudative AMD according to the international classification and grading system for age-related maculopathy and AMD^37^. Participants were categorized according to the findings in the worse eye. For atrophic AMD, only those with geographic atrophy, the advance AMD, were included, while those with mild to moderate dry AMD were excluded from the study. CNV was further divided into two angiographic subtypes: classic CNV and occult CNV, based on its appearance under fluorescein angiography^38^.

### Collection of blood samples and measurement of DKK-1

As described previously^12^, blood samples were drawn from the antecubital vein and collected into tubes containing heparin. After samples were centrifuged at 3000 RPM for 10 minutes at 4 °C, the plasma was separated and stored at −80 °C until the assay was performed. Plasma levels of DKK-1 were measured using a commercial enzyme-linked immunosorbent assay (ELISA) kits (R&D Systems, Minneapolis, MN, USA). As described previously^12^, the procedures were performed according to the instructions from the manufacturer. The person performing ELISA was blind to the information of samples. All of the measurements were performed in triplicate for each sample, and the mean values were calculated. Inter- and intra-assay variations were 4.1% and 6.0%, respectively.

### Statistical analysis

Statistical analyses were performed with SPSS (16.0). The variables distribution pattern was evaluated with one-sample Kolmogorov–Smirnov test. Data was presented as n (%), mean ± standard deviation (SD) and the interquartile ranges. Chi-square test was used to compare categorical variables. One-way ANOVA was performed for multiple comparisons, and post hoc Tamhane analysis and Student t-test were applied when only two independent variables were assigned. An unconditional logistic regression analysis was used to measure the odds ratios (OR) and 95% confidence intervals (CI) of DKK-1 levels between cases and the controls. Additionally, a receiver-operating characteristic analysis (ROC) was performed to obtain the ideal DKK-1 level cutoff score for differentiating patients from healthy controls. The optimal cut-off points of DKK-1 for the detection of exudative AMD were determined by Youden index J. The diagnostic accuracy was accessed by the area under the curve (AUC) with 95% CI, and sensitivity and specificity. *P* values < 0.05 were considered to be statistically significant.
